# Molecular hallmarks of cognitive resilience and resistance in Alzheimer's disease: Insights from excitatory and inhibitory neurons

**DOI:** 10.1002/alz70855_096285

**Published:** 2025-12-23

**Authors:** Isabel Castanho, Pourya Naderi Yeganeh, Carles A Boix, Sarah Morgan, Hansruedi Mathys, Dmitry Prokopenko, Bartholomew White, Larisa M. Soto, Giulia Pegoraro, Saloni Shah, Athanasios Ploumakis, Nikolas Kalavros, David A. A. Bennett, Christoph Lange, Doo Yeon Kim, Lars Bertram, Li‐Huei Tsai, Manolis Kellis, Rudolph E. Tanzi, Winston Hide

**Affiliations:** ^1^ Beth Israel Deaconess Medical Center, Boston, MA, USA; ^2^ Harvard Medical School, Boston, MA, USA; ^3^ Massachusetts Institute of Technology, Cambridge, MA, USA; ^4^ Queen Mary University of London, London, MA, United Kingdom; ^5^ University of Pittsburgh, Pittsburgh, PA, USA; ^6^ Massachusetts General Hospital, Charlestown, MA, USA; ^7^ University of Exeter, Exeter, United Kingdom; ^8^ Rush Alzheimer's Disease Center, Chicago, IL, USA; ^9^ Harvard T.H. Chan School of Public Health, Boston, MA, USA; ^10^ University of Lübeck, Lübeck, Germany; ^11^ Center for Lifespan Changes in Brain and Cognition (LCBC), Department of Psychology, University of Oslo, Oslo, Norway; ^12^ Broad Institute of MIT and Harvard, Cambridge, MA, USA; ^13^ Picower Institute for Learning and Memory, Cambridge, MA, USA

## Abstract

**Background:**

Cognitive resilience, the ability to maintain cognitive function despite extensive Alzheimer's disease (AD) pathology, offers a unique window into natural protective mechanisms. Understanding its molecular and cellular basis can reveal powerful therapeutic approaches for AD. This study investigates the molecular hallmarks of cognitive resilience and protection against AD by integrating genetic, transcriptomic, and cellular data.

**Method:**

We interrogated bulk RNA sequencing (*n* = 631) and single‐nucleus RNA sequencing (*n* = 48) data from multiple brain regions from the Religious Orders Study and the Memory and Aging Project (ROSMAP). Subjects were classified into AD, resilient, and control groups based on cognitive status and β‐amyloid and tau pathology. Genetic analyses were paired with transcriptomic profiling and cell population mapping to identify resilience‐associated pathways and protected neuronal subtypes.

**Results:**

Cognitive resilience was associated with an intermediate AD polygenic risk profile and subtle tissue‐level transcriptomic changes, including upregulation of *GFAP* and downregulation of *KLF4*. Investigations of cellular resilience revealed a reorganization of protein folding and degradation pathways in excitatory neurons. Critical resilient excitatory neuronal subpopulations mapped in the entorhinal cortex exhibited distinctive protective signaling through neurotrophin and angiopoietin pathways. Novel candidate markers of resistance and protection‐associated inhibitory neurons included RBFOX1 and KIF26B.

**Conclusion:**

This study reveals cellular and molecular features of cognitive resilience, highlighting the roles of excitatory and inhibitory neurons, protein folding pathways, and protective signaling networks. Our observations expose that the excitatory‐inhibitory balance, which is disrupted in AD, is maintained in resilience. Our findings provide a foundation for leveraging resilience mechanisms to develop targeted therapies for AD.

